# 

**Published:** 1919-02

**Authors:** 


### Texas Medical Journal

ARMY
MEDICAL
SEP 2 3 1947
LIBRARY

Entered at the postoffice at Austin, Texas, as second-class mail matter

